# A Time-Course Transcriptomic Resource for Early Embryogenesis in *Lethenteron reissneri*

**DOI:** 10.3390/ijms27209001

**Published:** 2026-10-11

**Authors:** Weiyin Xu, Jun Li, Guiyao Wang, Qingkui Bai, Huiyi Fu, Ji Zhao, Shuiqing Yu, Ting Zhu

**Affiliations:** 1 College of Life Sciences, Liaoning Normal University, Dalian 116081, China; 2 Lamprey Research Center, Liaoning Normal University, Dalian 116081, China

**Keywords:** Lamprey, jawless vertebrates, embryonic patterning, time-course transcriptome, temporal gene expression

## Abstract

Continuous developmental transcriptomic data remain limited for jawless vertebrates. We generated an RNA-seq time series spanning 2 h post-fertilization (hpf; fertilization stage) to 10 days post-fertilization (dpf; hatching stage) in *Lethenteron reissneri*, with 14 developmental stages and three biological replicates per stage. We examined global expression patterns, differential expression between adjacent stages, functional enrichment, and stage-level co-expression, and assessed representative expression trends using reverse transcription quantitative PCR (RT-qPCR). Gene-expression profiles changed progressively across development. One notable interval was 2.5–3 dpf (late blastula to early gastrula), when upregulated genes were broadly enriched for germ-layer formation, axial patterning, neural development, and morphogenesis rather than the RNA-processing, ribosome-biogenesis, and translation terms prominent at earlier stages. Stage-level co-expression analysis identified a module enriched for development-related terms, and differential expression, functional annotation, and temporal profiles were used to compile a list of 154 candidate developmental genes. This study provides a continuous transcriptomic resource for early *L. reissneri* embryogenesis and a temporal framework for subsequent studies of lamprey development.

## 1. Introduction

Many key events in early embryogenesis are reflected in gene expression changes before they become morphologically apparent [1,2]. Conventional morphological staging describes embryonic structures at successive stages, but morphology alone cannot determine when transcriptional programs begin to change, when those changes are concentrated, or when programs associated with germ-layer formation, axial patterning, and neural development emerge. By allowing adjacent stages to be compared directly, densely sampled transcriptomic time series can localize intervals of concentrated transcriptional change and relate them to the corresponding developmental events [3,4].

Time-series studies across vertebrates have revealed both conservation and lineage-specific variation in the timing of early developmental programs. Single-cell transcriptomes from human oocytes to morulae helped resolve sequential changes in modules associated with the cell cycle, gene regulation, translation, and metabolism [5]. Seven of nine human stage-specific modules were preserved in mice, although their stage specificity and timing were not identical [5]. In the rhesus macaque (*Macaca mulatta*), transcriptomic remodeling was greatest between the 4- and 8-cell stages, with major zygotic genome activation occurring at the 8-cell stage; overall transcriptional dynamics were more similar to those in humans than in mice [6,7]. In mice, an embryonic day (E) E6.5–E8.5 single-cell atlas, spanning gastrulation to early organogenesis, linked transcriptional changes during gastrulation to germ-layer diversification and the formation of major organ progenitors [1]. A single-cell time series in *Xenopus* spanning zygotic genome activation through early organogenesis reconstructed differentiation across the major embryonic lineages [8]. Comparison with a corresponding 3.3–12 h post-fertilization (hpf) *Danio rerio* (zebrafish) series, spanning the blastula to early segmentation stages, revealed both conserved and divergent features of early vertebrate gene expression programs [2,8]. At a broader scale, comparative transcriptomic analysis across mice, chicken, *Xenopus*, and zebrafish identified the pharyngula as the period of greatest transcriptomic similarity [9]. Together, these findings indicate that vertebrate embryos share conserved components of their developmental transcriptional programs, although the timing and boundaries of individual transitions vary among lineages.

Comparable time-series evidence remains sparse in jawless vertebrates, an early-diverging branch of vertebrate evolution. Continuous datasets are therefore needed to describe how gene expression changes across defined stages of lamprey development. Lampreys also provide an important comparative context for studies of vertebrate developmental programs. Piavis [10] established a developmental staging system for the sea lamprey, whereas Tahara [11] divided the development of *Lethenteron reissneri*, from the unfertilized egg to the pre-burrowing ammocoete, into 31 standard stages. Li et al. [12] subsequently described the early development of a closely related lamprey species on the basis of external morphology and histological features. These staging frameworks have underpinned studies of lamprey neural crest migration and its regulation [13,14]. *Lethenteron reissneri* is a freshwater lamprey native to Northeast Asia and occurs in China primarily in the Heilongjiang (Amur) River basin and northeastern drainages, including the Taizi River. Molecular resources for this species include a chromosome-level reference genome [15] and, more recently, a three-dimensional single-cell transcriptomic atlas of the whole brain [16]. Artificial fertilization and embryo culture also permit consecutive developmental stages to be sampled. Despite these staging, embryological, and genomic resources, continuous time-series data remain limited for describing the progression of gene-expression programs during early *L. reissneri* development.

We therefore generated an RNA-seq time series comprising 14 developmental stages from 2 hpf to 10 dpf in *L. reissneri* embryos. We used adjacent-stage differential expression and functional enrichment to describe changes across the series, and stage-level co-expression analysis to organize genes with similar temporal profiles. The data identify broad changes in developmental functions around 2.5–3 dpf and provide a temporal transcriptomic resource for subsequent studies of lamprey embryogenesis.

## 2. Results

### 2.1. RNA-Seq Dataset and Quality Assessment

**Figure 1:**
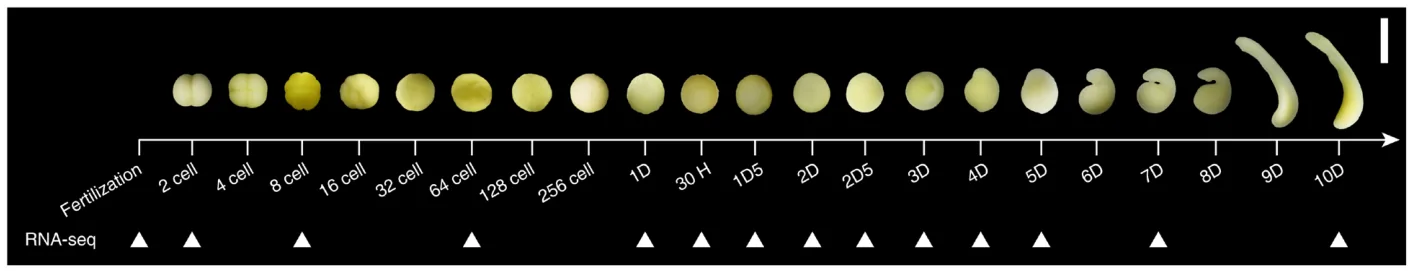
Time-course sampling and transcriptomic analysis framework for early embryonic development in *Lethenteron reissneri*. Representative embryo morphology and the developmental timeline from fertilization to 10 dpf, including early cleavage. White triangles indicate stages sampled for RNA-seq. Scale bar, 1000 μm. Corresponding developmental stages and degree-day values for all sampling points are listed in Table 1.

We generated an RNA-seq time series spanning 14 developmental stages from 2 hpf to 10 dpf, with three biological replicates per stage (42 samples in total). Sampling covered the early post-fertilization period, cleavage, gastrulation, neurulation, and pre-hatching stages. Representative embryo morphology and the RNA-seq sampling scheme are shown in Figure 1. The corresponding morphological stages and degree-day values are summarized in Table 1.

All 42 samples passed the pre-library quality assessment. Median RNA concentration, total RNA yield, and RNA quality number (RQN) were 39 ng/μL, 1.37 μg, and 6.35, respectively. Sequencing generated 394.54 Gb of raw data comprising 2,630,402,750 raw reads. After filtering, 372.21 Gb of clean data comprising 2,481,231,788 clean reads were retained. Q20 and Q30 values ranged from 95.32% to 97.40% and from 89.90% to 93.96%, respectively.

**Figure 2:**
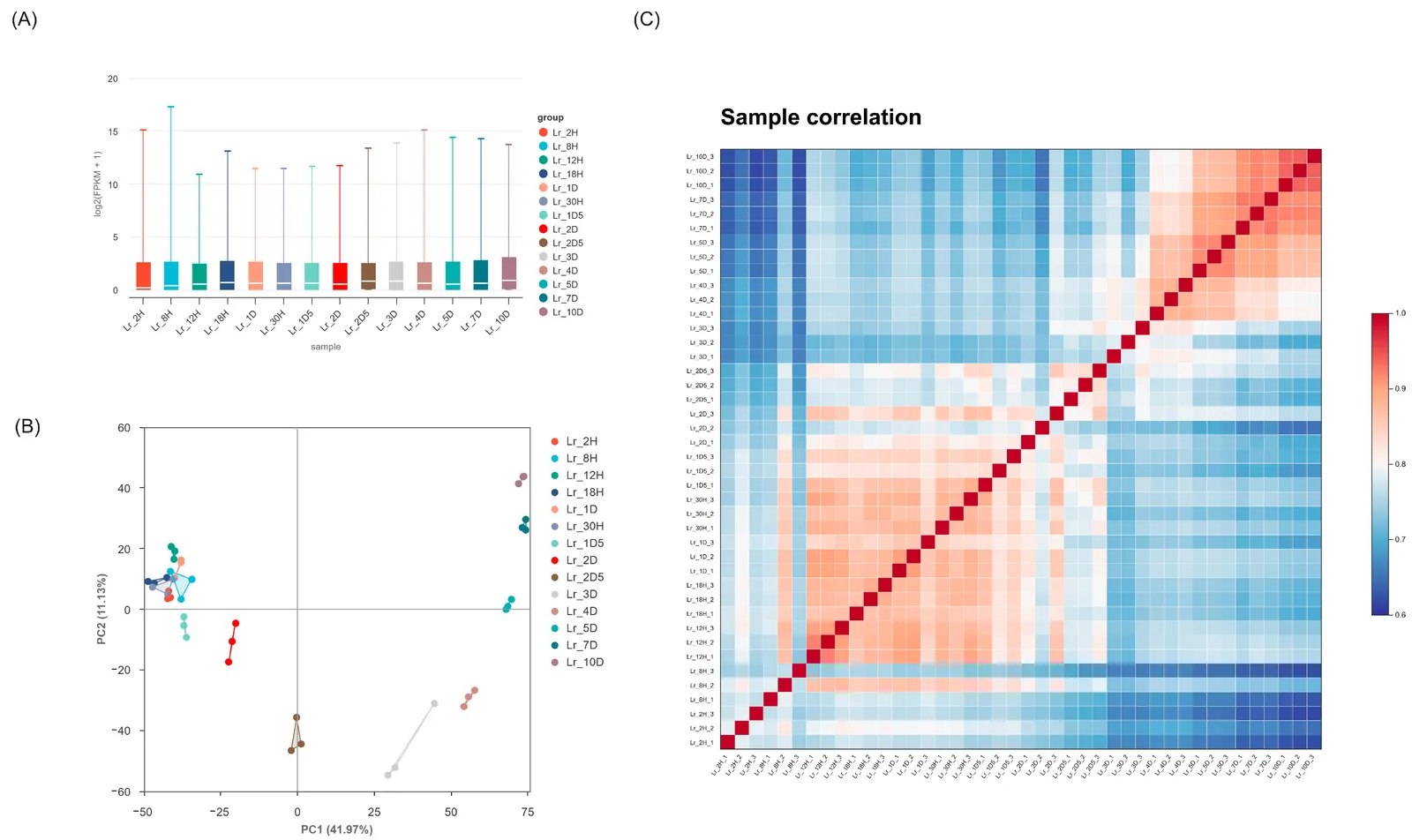
RNA-seq data quality and global expression structure. (**A**) Distribution of log_2_ (FPKM + 1) values among RNA-seq samples from each developmental stage. (**B**) Principal component analysis of the time-course transcriptomes, showing the distribution of samples by developmental stage. PC1 and PC2 explain 41.97% and 11.13% of the total variance, respectively. (**C**) Heatmap of pairwise Spearman correlations calculated from the log_2_-transformed expression matrix.

Following alignment of the clean reads to the *L. reissneri* reference genome, total mapping rates ranged from 53.86% to 82.80%, with a mean of 68.68%, whereas unique mapping rates ranged from 46.58% to 78.01%, with a mean of 63.74%. The distributions of log2 (FPKM + 1) expression values were broadly comparable among samples (Figure 2A), and no sample showed an obvious quality anomaly or an outlying expression distribution. The sample with the lowest total mapping rate remained consistent with its developmental-stage group in the global expression analyses. The range of mapping rates is also compatible with the current state of the genome assembly and annotation for this non-model species. All 42 samples were retained for expression-matrix construction and subsequent analyses. Detailed sequencing and alignment statistics are provided in Supplementary Table S1.

### 2.2. Global Expression Patterns Revealed Stage-Associated Transcriptional Changes

Spearman correlation coefficients among biological replicates within stages ranged from 0.758 to 0.941. Correlations across developmental stages followed a stage-associated pattern, with temporally adjacent samples generally showing greater similarity than samples separated by longer intervals (Figure 2C).

In the principal component analysis (PCA), PC1 and PC2 accounted for 41.97% and 11.13% of the total expression variance, respectively. Biological replicates from the same stage generally clustered together, and sample positions shifted progressively along PC1 from negative to positive values over developmental time. Samples from 2 hpf to 1.5 dpf were located predominantly on the negative side of PC1, whereas those from 3 to 10 dpf were located mainly on the positive side (Figure 2B).

Both the correlation structure and PCA showed a progressive stage-associated shift in the global embryonic transcriptome.

### 2.3. Differential Expression Between Adjacent Stages Varied over Development

Differential expression was assessed between adjacent stages across 1, 1.5, 2, 2.5, 3, 4, 5, 7, and 10 dpf. Across the eight comparisons, 15,526 significant differential expression events were detected, involving 9684 unique genes. Each comparison included both upregulated and downregulated genes.

**Figure 3:**
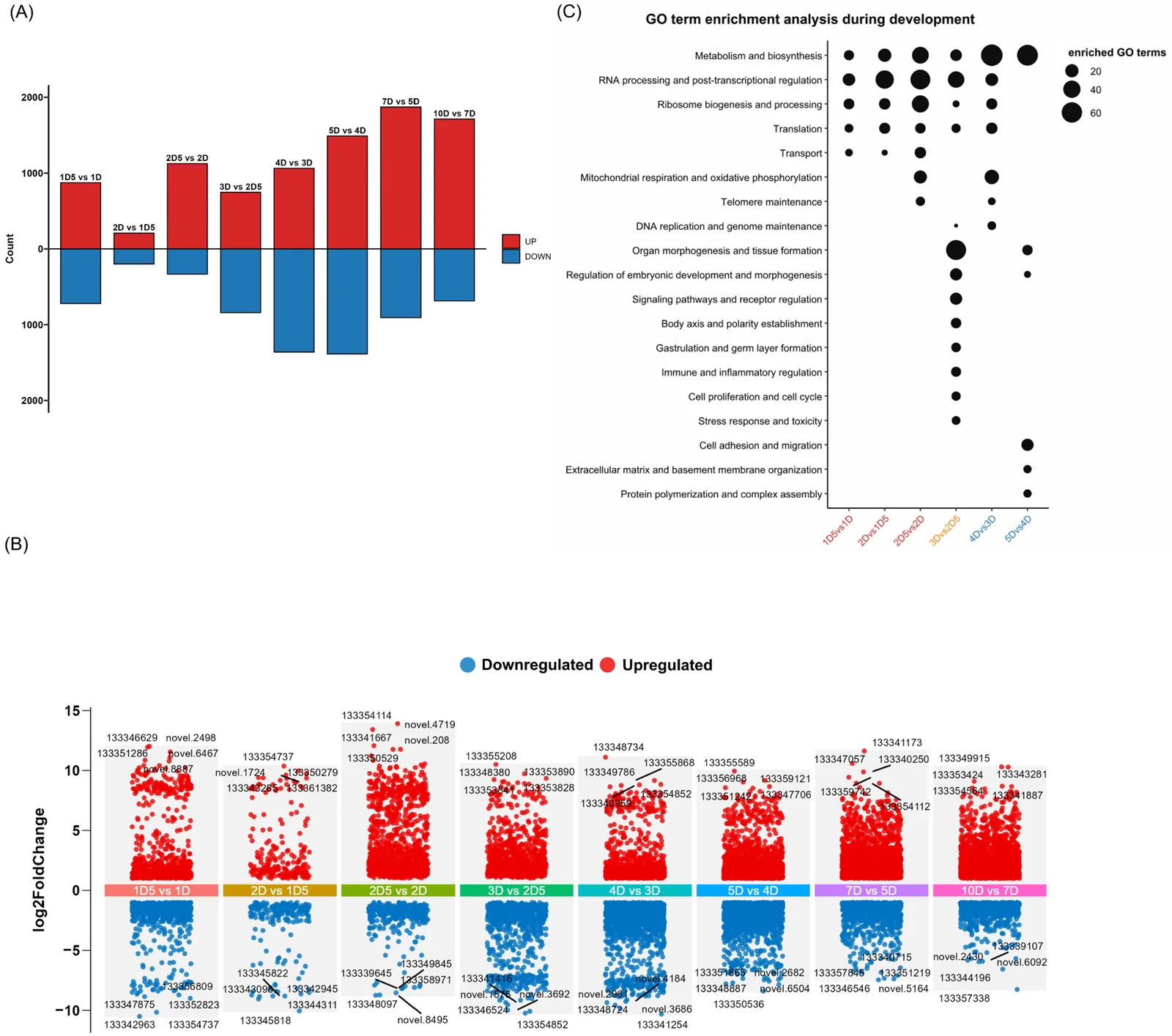
Differential expression and functional enrichment across adjacent developmental stages. (**A**) Numbers of upregulated and downregulated genes in adjacent-stage comparisons from 1 to 10 dpf. (**B**) Distribution of log2 fold changes among DEGs in each adjacent-stage comparison, with genes showing large-magnitude changes indicated. (**C**) Selected representative GO Biological Process categories enriched among genes upregulated in adjacent-stage comparisons.

The number of differentially expressed genes (DEGs) per comparison ranged from 408 to 2875. A total of 1594 DEGs were detected in the 1.5 dpf vs. 1 dpf comparison, including 873 upregulated and 721 downregulated genes. The smallest set was found in the 2 dpf vs. 1.5 dpf comparison, with 408 DEGs, of which 208 were upregulated and 200 were downregulated. The 2.5 dpf vs. 2 dpf and 3 dpf vs. 2.5 dpf comparisons yielded 1458 and 1588 DEGs, respectively. Subsequent comparisons identified 2425 DEGs in the 4 dpf vs. 3 dpf comparison, 2875 in the 5 dpf vs. 4 dpf comparison, 2778 in the 7 dpf vs. 5 dpf comparison, and 2400 in the 10 dpf vs. 7 dpf comparison (Figure 3A).

Using |log_2_FC| ≥ 2 to define large-magnitude changes, the eight comparisons contained 571, 176, 908, 965, 1111, 1219, 1202, and 1057 genes, respectively. These genes represented 35.8–62.3% of the DEGs in each comparison. The corresponding proportions were 62.3% for 2.5 dpf vs. 2 dpf and 60.8% for 3 dpf vs. 2.5 dpf (Figure 3B).

### 2.4. Gene Ontology (GO) Enrichment Across Adjacent Developmental Stages

Genes upregulated in each adjacent-stage comparison were subjected to GO Biological Process enrichment analysis. Significant terms in the 1.5 dpf vs. 1 dpf, 2 dpf vs. 1.5 dpf, and 2.5 dpf vs. 2 dpf comparisons were dominated by RNA processing, ribosome biogenesis, translation, metabolism and biosynthesis, and molecular transport. The 2.5 dpf vs. 2 dpf comparison additionally contained 19 terms related to mitochondrial respiration and oxidative phosphorylation and seven terms related to telomere maintenance.

In the 3 dpf vs. 2.5 dpf comparison, 107 development-related terms were distributed among categories related to signaling pathway and receptor regulation, embryonic development and morphogenesis, axis and polarity establishment, gastrulation and germ-layer formation, and organ morphogenesis and tissue development. These comprised 16 terms related to signal regulation, 16 related to embryonic development, 10 related to axis and polarity establishment, eight related to gastrulation and germ-layer formation, and 57 related to organ morphogenesis and tissue development. The represented processes included regulation of Wnt and BMP signaling, germ-layer formation, axial patterning, neural development, and morphogenesis of multiple organs. Because GO terms are hierarchical and frequently overlap, these counts summarize the functional breadth represented in the enrichment results.

In the 4 dpf vs. 3 dpf comparison, significant terms were dominated by metabolism and biosynthesis (68 terms) and mitochondrial respiration and oxidative phosphorylation (25 terms), together with processes involving RNA processing, ribosome biogenesis, translation, DNA replication, and genome maintenance. In the 5 dpf vs. 4 dpf comparison, 63 terms were related to metabolism and biosynthesis and 16 to cell adhesion and migration; additional terms involved organ morphogenesis, extracellular matrix and basement membrane organization, and protein-complex assembly.

Overall, the enrichment profiles varied across development. Figure 3C summarizes selected representative GO Biological Process categories rather than providing a formal ranking of developmental intervals. Upregulated genes at earlier stages were dominated by RNA processing, translation, metabolism, and biosynthesis. The 3 dpf vs. 2.5 dpf comparison showed broad enrichment in developmental signaling, germ-layer formation, axial patterning, and organ morphogenesis, whereas the 4–5 dpf comparisons were dominated by metabolism, genome maintenance, cell adhesion, and extracellular matrix-related processes (Figure 3C).

### 2.5. Stage-Level Co-Expression Analysis of the Red Module

**Figure 4:**
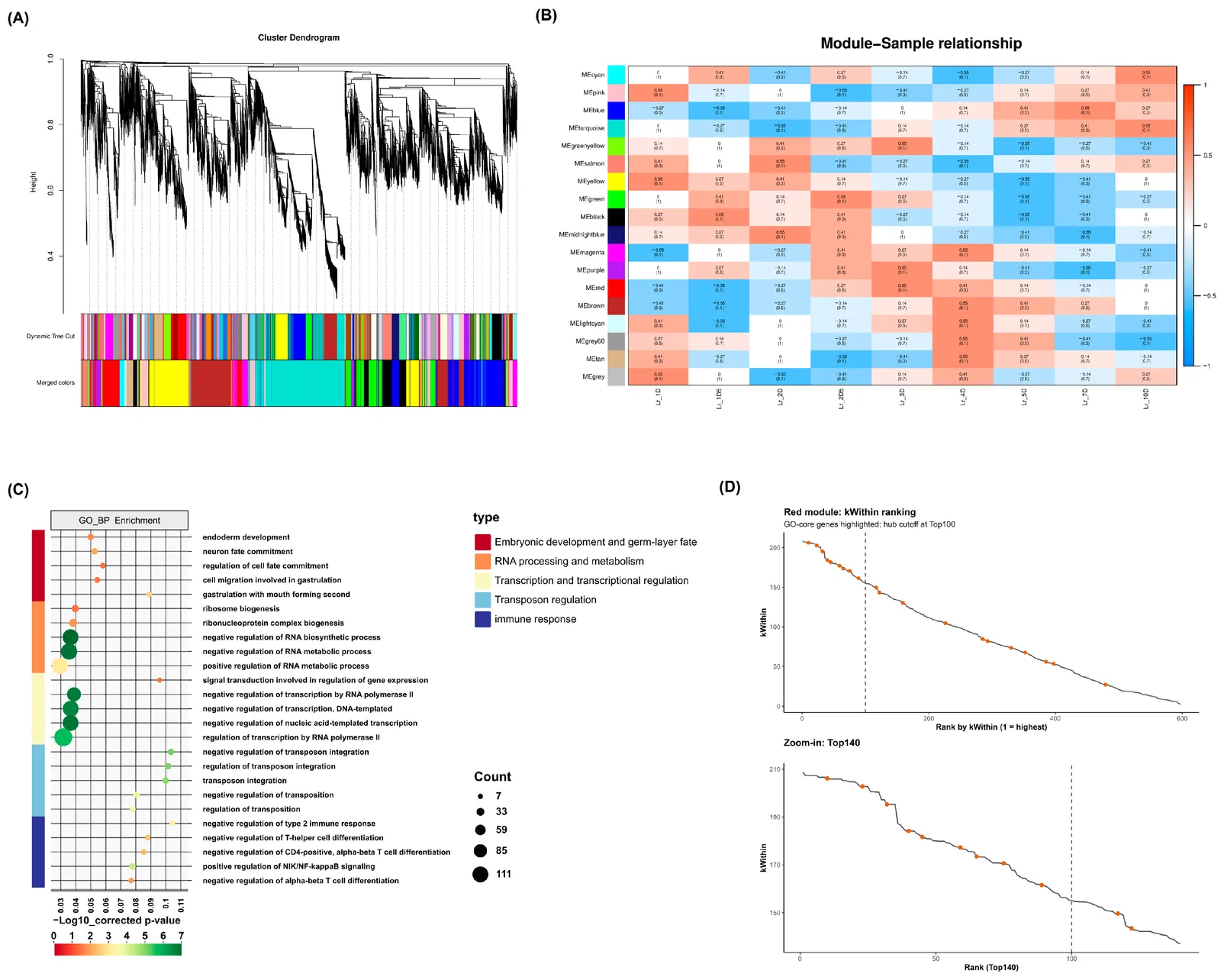
Stage-level WGCNA [17] and functional characteristics of the red module. Expression values from 27 samples spanning 1–10 dpf were averaged within developmental stages to obtain nine stage-mean expression profiles, and a WGCNA network was constructed from 12,185 genes. (**A**) Gene dendrogram with initial module assignments from Dynamic Tree Cut [18] and final assignments after module merging. (**B**) Heatmap of correlations between module eigengenes and the nine stage-mean profiles. (**C**) GO Biological Process enrichment of the red module. (**D**) Overlap between GO-core genes and the top-100 high-connectivity gene set.

For the 27 samples representing nine stages from 1 to 10 dpf, expression values were averaged across the three biological replicates at each stage, yielding nine stage-mean expression profiles. A stage-level co-expression network was constructed from the 12,185 retained genes and resolved into 17 co-expression modules; genes not assigned to a specific module were placed in the grey set and were not counted as a module. Soft-threshold selection is shown in Supplementary Figure S1, and gene clustering and module assignments are shown in Figure 4A. The network topological overlap heatmap displayed the resulting module structure (Supplementary Figure S2).

The module eigengenes showed different relationships with the stage-mean profiles (Figure 4B). The red, purple, and magenta modules clustered together in the module eigengene dendrogram (Supplementary Figure S3). Comparison of functional enrichment across all modules identified 136 significant GO Biological Process terms in the red module (FDR ≤ 0.05), including 16 development-related terms; the remaining modules did not yield large numbers of significant terms at the same threshold. Based on this module-wide comparison, subsequent analyses focused on the red module.

The red module contained 597 genes and was significantly enriched for developmental processes including endoderm development, neuron fate commitment, regulation of cell fate commitment, gastrulation, and cell migration involved in gastrulation. Other enriched processes included RNA biosynthesis and metabolism, transcriptional regulation, transposon regulation, and immune responses (Figure 4C).

Threshold sensitivity analysis identified 42, 31, 20, and 17 candidate genes at term-count thresholds of ≥2, ≥3, ≥4, and ≥5, respectively; 13, 10, nine, and seven of these genes overlapped with the top-100 high-connectivity gene set in the red module. At the ≥4 threshold, 45.0% of the candidate genes were included in the top-100 high-connectivity gene set, with a fold enrichment of 2.69; both values were the highest among the thresholds evaluated. Accordingly, the ≥4 threshold was used as the operational criterion for GO-core gene prioritization. The final set comprised 20 GO-core genes, including *CDH2*, *TBX20*, *LEFTY1*, *FGF8*, *NODAL*, *TBXT*, and *SMAD4*; the complete list is provided in Supplementary Table S2.

Nine of the 20 GO-core genes were present in the top-100 high-connectivity gene set of the red module, compared with a random expectation of 3.35 genes (fold enrichment = 2.69; nominal one-sided hypergeometric *p* = 0.00246; Figure 4D). A red-module subnetwork constructed from the 44 unique genes associated with five development-related GO terms retained 805 of the 946 possible gene pairs at a connection-weight threshold of 0.02, showing broad connectivity among the selected genes (Supplementary Figure S4).

### 2.6. Kyoto Encyclopedia of Genes and Genomes (KEGG) Pathway Mapping of Genes Upregulated at 3 dpf

The set of genes upregulated at 3 dpf relative to 2.5 dpf was mapped using KEGG Mapper [19]. The mapped genes were distributed across multiple regulatory levels of the MAPK, Notch, TGF-β, and Wnt signaling pathways (Supplementary Figure S5).

Mapped nodes in the MAPK pathway included *GF/RTK*, *MKK/JNK/MKP*, and *c-Fos/AP-1* components. Notch pathway nodes included *Delta*, *Dvl*, *Hes*, *NRARP*, and *Gro/TLE*. In the TGF-β pathway, mapped nodes included *FST*, *THBS1*, *BMPR*, *Activin/Nodal* receptors, *Smad6/7*, *BAMBI*, *Id*, and *RhoA*. Wnt pathway nodes included *R-spondin*, *Wnt*, *Frizzled*, *LRP5/6*, *Axin*, and *Nkd*, together with *c-Jun*, *WISP1*, and planar cell polarity components such as *Prickle*, *Daam1*, *RhoA*, and *JNK*.

### 2.7. Functional Classification and Temporal Expression Patterns of Candidate Developmental Genes

To describe gene-level changes during this developmental period, we focused on genes related to developmental processes. The criteria described above for differential expression, red-module GO-core status, developmental annotation, and temporal expression were used to compile a set of 154 candidate developmental genes. Of these, 56 were assigned to signaling categories including Wnt, BMP/TGF-β, FGF/RTK–MAPK, Notch, and Hedgehog, and 46 were associated with germ-layer formation, axial patterning, and regional identity. Another 15 genes were associated with neural differentiation, neural crest development, and axon guidance; 17 with organ and tissue progenitors; and 20 with cell migration, cytoskeletal remodeling, and morphogenesis. Candidate genes, functional classifications, and the basis for their inclusion are listed in Supplementary Table S3.

**Figure 5:**
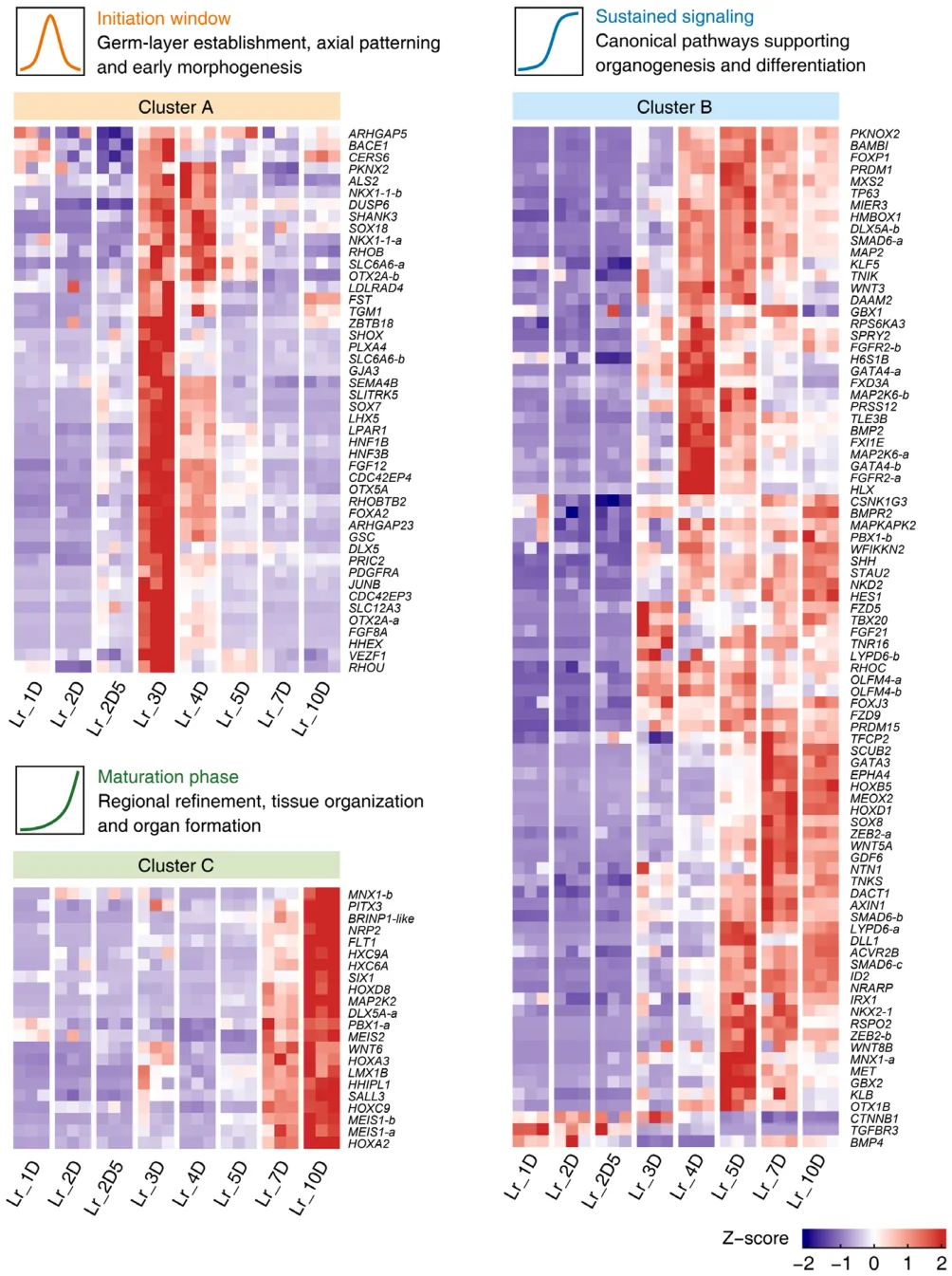
Temporal expression patterns of candidate developmental genes associated with the 3 dpf vs. 2.5 dpf comparison. Candidate genes were compiled using upregulation in the 3 dpf vs. 2.5 dpf comparison, development-related functional annotations, pathway membership, and WGCNA/GO-core information. They were divided into three clusters according to the timing of their main expression increase and their subsequent expression trajectory. Cluster A genes increased mainly at 3–4 dpf and subsequently declined. Cluster B genes increased from 3 dpf and remained relatively highly expressed at later stages. Cluster C genes increased primarily at 7–10 dpf. Expression values were standardized within each gene using row-wise Z-scores; colors indicate relative standardized expression. Gene symbols are shown where available; lowercase suffixes (a, b, or c) distinguish separate *L. reissneri* loci assigned the same gene symbol.

The temporal expression profiles were divided into three clusters according to when their main expression increase occurred and how expression changed afterward. Cluster A contained 46 genes whose expression increased predominantly at 3–4 dpf and subsequently declined; representative genes included *FOXA2*, *GSC*, *HHEX*, *OTX2A*, and *SOX7*. Cluster B contained 86 genes whose expression increased from 3 dpf and remained relatively high at subsequent stages, including *WNT5A*, *DACT1*, *AXIN1*, *DLL1*, *BMPR2*, *BAMBI*, *SMAD6*, and *NRARP*. Cluster C contained 22 genes whose expression increased mainly at 7–10 dpf, including *HOXA2*, *HOXA3*, *MEIS1*, *MEIS2*, *PBX1-b*, *LMX1B*, *WNT6*, and *SALL3*. The three clusters differed in the onset, peak stage, and duration of elevated expression (Figure 5).

### 2.8. RT-qPCR Validation of Representative Gene Expression Trends

To assess the temporal expression trends obtained by RNA-seq, eight representative genes were selected for RT-qPCR on the basis of differential expression, functional class, co-expression module membership, and temporal profile. RT-qPCR was performed at 1, 2, 2.5, 3, 4, 5, 7, and 10 dpf. The selected genes spanned several functional classes and temporal profiles, including red-module members, genes associated with Notch and MAPK signaling, and homeobox transcription factors. Their RNA-seq profiles encompassed increases around 2.5–3 dpf, sustained high expression thereafter, and gradual increases during later development.

The reference gene was selected from the RNA-seq expression matrix across all developmental stages. LOC133343421 (*ATP5MK*) had a mean expression of 496.61 FPKM and a standard deviation of 0.36 after log_2_ (FPKM + 1) transformation, indicating limited variation across the time series; it was therefore used for RT-qPCR normalization. Across the 24 biological samples, its Ct values ranged from 22.88 to 23.30, with a mean of 23.08 and a CV of 0.49%, indicating limited variation across the developmental stages examined.

**Figure 6:**
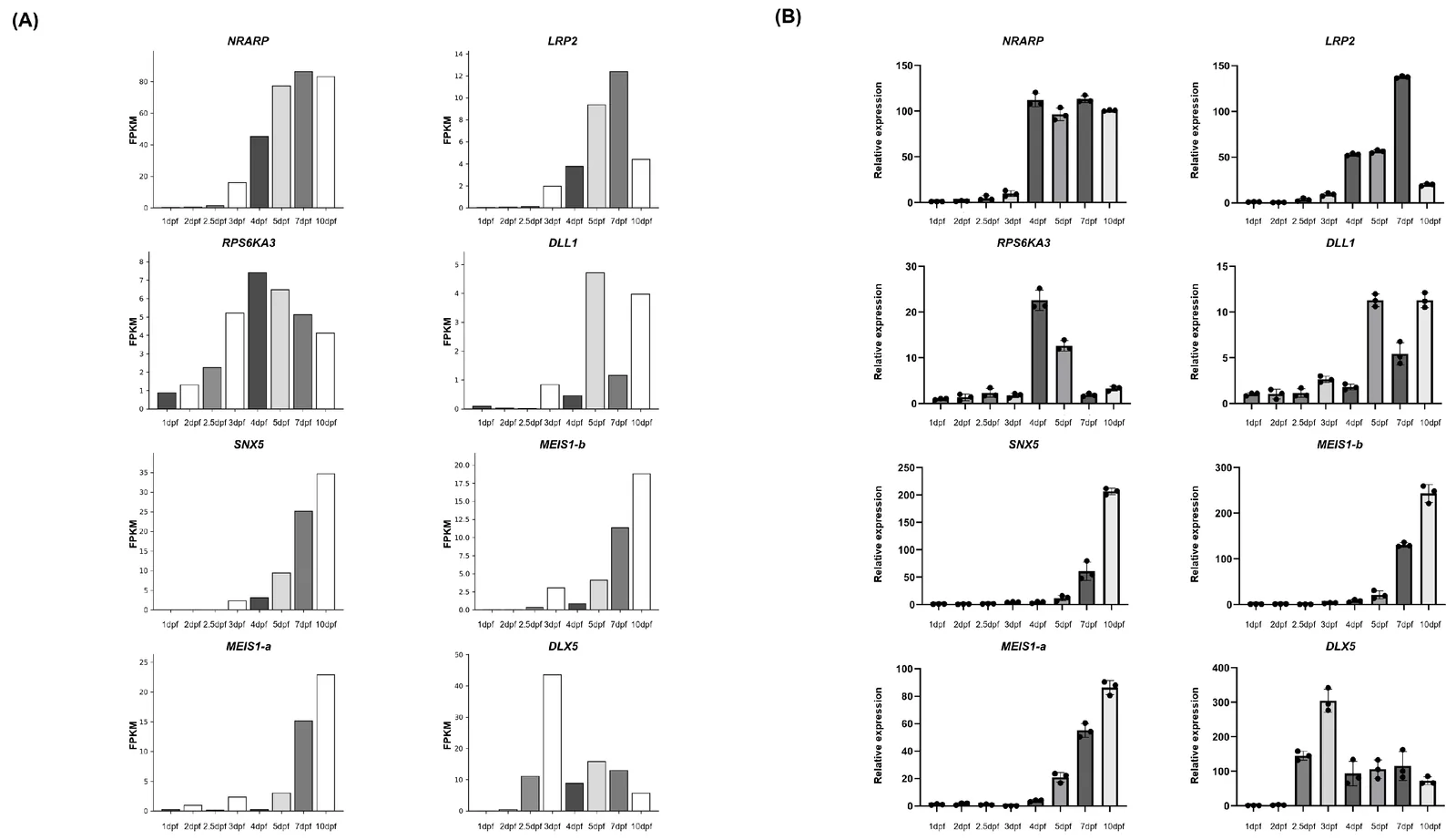
RT-qPCR assessment of candidate-gene expression trends. Eight candidate genes were analyzed by RT-qPCR at eight developmental stages from 1 to 10 dpf. (**A**) RNA-seq expression profiles of the candidate genes; FPKM values are means of three biological replicates per stage. (**B**) RT-qPCR expression profiles of the candidate genes. Relative expression was normalized to *ATP5MK* (LOC133343421) and calculated using the 2^−ΔΔCt^ method. Three independent biological replicates were analyzed for each gene at each developmental stage, with three technical replicate wells per biological replicate. Ct values were first averaged across technical replicates within each biological replicate, after which relative expression was calculated separately for each biological replicate. Bars show means, and error bars indicate standard deviations (SDs). Gene symbols are shown above each panel; *MEIS1-a* and *MEIS1-b* distinguish two separate *L. reissneri* loci annotated as *MEIS1*.

For all eight genes, the RT-qPCR and RNA-seq profiles showed concordant overall trends and similar periods of increased expression (Figure 6A,B). This concordance provides experimental support for the temporal expression patterns identified from the RNA-seq data.

## 3. Discussion

### 3.1. Temporal Changes in Developmental Programs

The 14-stage time series describes the progression of gene expression from fertilization to hatching in *Lethenteron reissneri*. Across the series, the functional profile of upregulated genes changed from RNA processing, ribosome biogenesis, and translation at earlier stages to a broader representation of developmental processes at later stages. The late-blastula-to-early-gastrula interval (2.5–3 dpf) was notable for enrichment related to germ-layer formation, axial patterning, neural development, and morphogenesis. Previous lamprey studies have largely focused on specific tissues, individual developmental events, or a small number of representative stages, whereas the continuous series examined here describes these changes across consecutive developmental stages. The transcriptomic data were generally consistent among biological replicates and provide a basis for examining temporal expression patterns during early development.

Adjacent-stage analysis identified 1588 DEGs in the 3 dpf vs. 2.5 dpf comparison, including 965 genes with |log_2_FC| ≥ 2. Both the number of DEGs and the proportion of large expression changes increased after 2.5 dpf, although several later comparisons contained more DEGs. The prominence of the 2.5–3 dpf interval in our interpretation reflects the developmental functions represented among its upregulated genes.

Genes upregulated at 3 dpf relative to 2.5 dpf were enriched for gastrulation, germ-layer formation, axis and polarity establishment, cell fate determination, neural development, and organ morphogenesis. This broad functional profile places these developmental changes within the late-blastula-to-early-gastrula interval. We interpret this period as a concentration of development-related expression changes rather than as a statistically discrete change point.

The time series also resolved the temporal sequence of functional changes surrounding this period. Genes upregulated at earlier stages were predominantly associated with RNA processing, ribosome biogenesis, translation, metabolism, and biosynthesis. Mitochondrial respiration and oxidative phosphorylation became more prominent around 2.5 dpf, whereas at 3 dpf, enrichment centered on terms associated with germ layers, body axes, neural development, and morphogenesis. After 4 dpf, enrichment shifted toward DNA replication and genome maintenance, metabolism, cell adhesion, extracellular matrix organization, and cytoskeletal remodeling. Time-course studies in zebrafish, amphioxus, and chicken embryos have similarly distinguished the expression states of adjacent stages and localized functional changes to relatively discrete developmental intervals [3,4,20,21]. Although the absolute timing differs among species, these studies illustrate the temporal organization of functional transitions during early development.

Existing embryological and transcriptomic studies provide developmental context for the 2.5–3 dpf interval in lampreys. Tahara [11] established a continuous staging series for *L. reissneri*. At the same culture temperature used in the present study (18 ± 1 °C), Li et al. [12] defined the blastula and gastrula periods of a closely related lamprey as 22–70 hpf and 3–5 dpf, respectively, placing the present interval near the transition from late blastula to early gastrula. The germ-layer-related genes *brachyury*, *soxF*, and *foxA2* also begin to be expressed during early gastrulation in lampreys [22]. In sea lamprey, the number of transcriptionally active genes increases after 2.5 dpf, a change linked to the onset of zygotic transcription [23]. Together, the staging literature and enrichment results place the observed expression changes near the late-blastula-to-early-gastrula progression.

### 3.2. Development-Related Pathways and Temporal Gene Programs

In the 3 dpf vs. 2.5 dpf comparison, upregulated genes were broadly distributed across the Wnt, TGF-β/BMP, Notch, and MAPK developmental signaling pathways. Wnt-related changes involved RSPO/WNT signaling inputs, FZD/LRP receptor complexes, intracellular regulators including *DACT1*, *AXIN1*, and *NKD2*, downstream transcriptional responses, and the noncanonical planar cell polarity branch. TGF-β/BMP-related changes encompassed extracellular regulators, BMPR/Activin/Nodal receptors, SMAD signaling components, and negative-feedback regulators including *BAMBI*, *SMAD6*, and *SMAD7*. Changes in Notch- and MAPK-related genes likewise spanned ligand–receptor recognition, intracellular signal transduction, nuclear responses, and feedback regulation. Developmental regulators closely associated with germ-layer differentiation, axial patterning, and regional identity, including *NODAL*, *LEFTY1*, *SMAD4*, *TBXT*, *FOXA2*, *OTX2*, and *SOX7*, were also upregulated. These findings indicate that multiple signaling pathways and developmental regulators showed coordinated expression changes during this period, reflecting broad transcriptional reorganization rather than changes restricted to a single pathway.

These observations are broadly consistent with previous studies of early developmental regulation in lampreys. FGF, Wnt, and Hedgehog signaling contribute to regional patterning of the lamprey brain [24], whereas the neural crest gene regulatory network is involved in cell fate determination and migration [14,25]. BMP-, Nodal-, and Wnt-related factors contribute to axial induction [26], and retinoic acid signaling is closely associated with hindbrain segmentation [27]. These studies indicate that early lamprey patterning and regional differentiation depend on the coordinated activity of multiple developmental signaling systems. Notably, the present study detected increases not only in components involved in signal input and transduction but also in inhibitory or feedback regulators, including *AXIN1*, *DACT1*, *BAMBI*, *SMAD6*, *FST*, and *NRARP*. The concurrent expression of signaling components and feedback regulators is consistent with temporal coordination of these systems during early patterning.

Stage-level WGCNA assigned many development-related genes to the red module. Overall expression of this module was elevated from approximately 2.5 to 4 dpf, and its annotations included gastrulation, endoderm development, neuron fate commitment, and regulation of cell fate commitment. The module also contained signaling regulators, intracellular transducers, and transcription factors associated with germ-layer formation and regional identity. These module-level patterns complement the adjacent-stage differential-expression and functional-enrichment results and provide a framework for organizing genes with similar temporal profiles.

Within the red module, nine of the 20 GO-core genes were included in the top-100 high-connectivity gene set, corresponding to a 2.69-fold enrichment and a nominal *p* value of 0.00246. The set included known developmental regulators such as *NODAL*, *LEFTY1*, *FGF8*, *SMAD4*, *TBXT*, *FOXA2*, *SOX7*, and *OTX2*. These genes provide useful reference points for interpreting the temporal and co-expression context of early *L. reissneri* embryogenesis. Together with the other candidates, they define a focused set for subsequent spatial-expression and functional studies.

The temporal profiles in Figure 5 organize the candidate genes by their subsequent expression trajectories. Cluster A genes increased transiently at 3–4 dpf and then declined. Cluster B genes increased from 3 dpf onward and remained at relatively high levels across several later stages, whereas Cluster C genes showed lower expression at early stages and increased predominantly at 7–10 dpf. These profiles distinguish transient, sustained, and later increases within the candidate set.

The functions represented by the three temporal patterns are consistent with this interpretation. Cluster A included *FOXA2*, *GSC*, *HHEX*, *OTX2A*, and *SOX7*, regulators associated with germ-layer formation, axial patterning, and early regional identity; their transient increase is consistent with the rapid establishment of early cell fates and positional information. Cluster B included signaling and feedback components such as *WNT5A*, *DACT1*, *AXIN1*, *DLL1*, *BMPR2*, *BAMBI*, *SMAD6*, and *NRARP*; their sustained expression is consistent with continued involvement of these signaling systems in subsequent tissue differentiation and morphogenesis. Cluster C genes, including *HOXA2*, *HOXA3*, *MEIS1*, *MEIS2*, *PBX1*, *LMX1B*, and *WNT6*, increased mainly at later stages and are more closely associated with the refinement of regional identity, tissue remodeling, and organ formation. High-resolution developmental atlases in zebrafish, *Xenopus*, and mice have similarly shown that transcriptional states established during gastrulation subsequently diverge along distinct cell-fate and tissue-development trajectories [1,2,8]. Lamprey Hox genes also exhibit dynamic segmental expression, and regulatory relationships among *Hox2*, *Meis*, and *Pbx* are deeply conserved across jawless and jawed vertebrates [28,29], providing comparative developmental context for the association of Cluster C with later regionalization. RT-qPCR analysis of eight candidate genes broadly reproduced the principal expression changes observed by RNA-seq, further supporting the temporal patterns described above.

### 3.3. Limitations and Future Directions

Lamprey-specific genome dynamics provide additional context for understanding the 2.5–3 dpf interval. During early sea lamprey embryogenesis, programmed genome rearrangement removes approximately 20% of the germline genome from somatic cell lineages and is largely complete by approximately 3 dpf [30,31]. More recent work showed that approximately 30% of the early sea lamprey genome undergoes a shift from maternal to paternal DNA methylation patterns and that sequences destined for programmed elimination are specifically hypermethylated before rearrangement begins [32].

In sea lamprey, these events occur at a time broadly similar to the pronounced transcriptional changes observed at 2.5–3 dpf in the present study. Because the studies involve different species and data types, a direct connection cannot currently be established; nevertheless, this temporal overlap raises the testable question of whether the transcriptional changes observed at 2.5–3 dpf in *L. reissneri* are also accompanied by changes in genome structure or epigenetic state. Joint measurements of DNA elimination, DNA methylation, chromatin accessibility, and transcription across the same developmental series could help determine whether these processes are related.

Several features define the scope of interpretation. Whole-embryo bulk RNA-seq does not resolve the cellular or spatial origin of expression changes, and the current *L. reissneri* genome annotation may influence mapping and functional assignments. The stage-level WGCNA summarizes broad temporal co-expression across nine stage-mean profiles; module stability and connectivity can be examined further in larger independently sampled time series. GO enrichment, GO-core screening, and candidate compilation form a connected prioritization workflow because they share functional annotations. Adjacent-stage intervals ranged from 0.5 to 3 days, and all samples developed at a nominal incubation temperature of 18 °C. The degree-day values in Table 1 facilitate comparison with other studies, while the effects of alternative temperature regimes remain to be tested.

Future studies combining standardized morphological staging, in situ hybridization, single-cell or spatial transcriptomics, and functional analyses of selected genes could resolve the cellular origins, spatial distribution, and regulatory relationships suggested by this time series. Overall, the study provides a continuous transcriptomic framework for early lamprey development, describes stage-associated changes in biological functions, and offers a candidate-gene list for subsequent investigation.

## 4. Materials and Methods

### 4.1. Broodstock, Artificial Fertilization, Embryo Culture, and Sampling

*L. reissneri* broodstock were reared and provided by the Lamprey Research Center of Liaoning Normal University. Before the experiment, broodstock were screened by gross external examination; all individuals used in the experiment displayed normal swimming and responsiveness, an intact body surface, and no visible lesions, hemorrhage, fungal growth, or other external abnormalities. During the breeding season, sexually mature females and males were selected on the basis of secondary sexual characteristics and gonadal maturity and then anesthetized by immersion in 0.01% MS-222 (tricaine methanesulfonate; Sigma-Aldrich, St. Louis, MO, USA; Cat. No. E10521). Mature eggs were gently stripped from the female abdomen into a beaker containing approximately 300 mL of culture water; milt was immediately stripped from a male into the same beaker and gently mixed with the eggs. The time at which the gametes were mixed was designated as 0 hpf. Fertilized eggs were rinsed three to five times with culture water and transferred to hatching tanks. The hatching tanks were 50 × 50 × 50 cm glass aquaria filled to a water depth of approximately 40 cm and lined with an approximately 1.5 cm layer of aquaculture-grade sand. Embryos were maintained under static-water conditions with continuous aeration using an air pump. Water temperature was maintained at 18 ± 1 °C, dissolved oxygen at ≥5.0 mg/L, and pH at 7.0 ± 0.2. Tap water aerated for at least 3 d was used as the culture water. Approximately one-third of the tank volume was replaced daily with temperature-matched water. The culture room was kept quiet and dimly lit, and dead or abnormally developing embryos were removed promptly. All procedures involving broodstock and embryos were approved by the Ethics Committee of Liaoning Normal University (protocol code LL2026202; approved on 13 August 2026) and were conducted in accordance with the approved institutional guidelines for animal care and use. The study is reported in accordance with the ARRIVE guidelines 2.0 [33].

Developmental stages were assigned using both time after fertilization and external embryonic morphology, with reference to established lamprey staging criteria [11,12]. A fertilization batch was considered to have reached a given stage when at least 70% of its embryos displayed the corresponding morphological features. At each sampling time, individual embryos were examined under a Nikon SMZ18 stereomicroscope (Nikon, Tokyo, Japan). Dead, damaged, or abnormally developing individuals were excluded, and morphologically normal embryos at comparable developmental states were pooled. Samples were collected at 2, 8, 12, 18, 24, and 30 hpf and at 1.5, 2, 2.5, 3, 4, 5, 7, and 10 dpf, representing 14 stages. Each of the three biological replicates was generated through independent egg and milt collection, fertilization, and embryo culture procedures. At each stage, 50 embryos from each of the three independent fertilization batches were pooled to form one biological replicate per batch, yielding 42 RNA-seq samples. After residual culture water was removed, each sample was immediately immersed in 1 mL RNAiso Plus (TaKaRa Bio, Kusatsu, Japan; Cat. No. 9108), snap-frozen in liquid nitrogen, and stored at −80 °C until RNA extraction. To provide an auxiliary temperature-referenced measure of developmental time, cumulative degree-days (°C·d) were calculated from fertilization as 18 °C × elapsed time (d). Accordingly, 2.5 and 3 dpf corresponded to 45 and 54 °C·d, respectively. Because a species-specific lower developmental threshold has not been established, these values are presented as a descriptive thermal-time index. A stage-level summary of sampling times, degree-day values, and corresponding morphological stages is provided in Table 1. A pool of 50 embryos collected from one independent fertilization batch at one developmental stage constituted one experimental unit. The study did not include treatment or control groups because it was designed as an observational developmental time course; therefore, random allocation to experimental groups was not applicable. Investigators were aware of developmental-stage labels during sampling and analysis because developmental stage was the variable of interest. All 42 RNA-seq libraries passed quality control and were retained for analysis.

### 4.2. RNA Extraction, Library Construction, and Sequencing

Total RNA extraction, quality assessment, library construction, and sequencing of the 42 embryo samples were performed by Novogene Co., Ltd. (Tianjin, China). Total RNA was extracted with RNAiso Plus (TaKaRa Bio, Kusatsu, Japan; Cat. No. 9108) according to the manufacturer’s instructions. RNA concentration and total yield were measured using a NanoDrop spectrophotometer (Thermo Fisher Scientific, Wilmington, DE, USA); the median concentration and yield were 39 ng/μL and 1.37 μg, respectively. RNA integrity and fragment-size distribution were assessed using an Agilent 5400 Fragment Analyzer (Agilent Technologies, Santa Clara, CA, USA), and the median RQN was 6.35. All samples received a “Pass” rating in the overall quality assessment and were subsequently used for library construction.

Poly (A)-containing mRNA was enriched using oligo (dT) magnetic beads and fragmented in NEB Fragmentation Buffer. First-strand cDNA was synthesized using random oligonucleotide primers and M-MuLV reverse transcriptase, followed by second-strand synthesis with RNase H and DNA polymerase I. Double-stranded cDNA was end-repaired, A-tailed at the 3′ ends, ligated to sequencing adapters, and PCR-amplified. AMPure XP beads (Beckman Coulter, Brea, CA, USA) were used to select libraries with inserts of approximately 250–300 bp.

Libraries were initially quantified using a Qubit 2.0 Fluorometer (Invitrogen, Carlsbad, CA, USA), and insert sizes were assessed using an Agilent 2100 Bioanalyzer (Agilent Technologies, Santa Clara, CA, USA). Effective library concentrations were determined by qPCR. Libraries that passed quality control were pooled according to the target data yield and sequenced on an Illumina high-throughput platform to generate 150-bp paired-end reads (PE150).

### 4.3. Sequencing Data Quality Control, Reference Genome Alignment, and Gene Expression Quantification

Raw paired-end reads were obtained in FASTQ format. Quality control was performed using fastp v0.19.7 [34] with the parameters -g -q 5 -u 50 -n 15 -l 150. Adapter sequences and poly-G tails were removed, and reads were discarded if they contained more than 15 ambiguous (N) bases, if more than 50% of bases had Phred quality scores below 5, or if the post-filtering read length was less than 150 bp. Base error rates, Q20, Q30, and GC content were calculated for the clean reads, which were then used in subsequent analyses.

Clean reads were aligned to the NCBI *L. reissneri* reference genome assembly GCF_015708825.1 (ASM1570882v1) [15] using HISAT2 v2.0.5 [35]. The genome index was built with HISAT2, and alignment was performed using default parameters. StringTie was used to assemble transcripts and predict putative novel gene models [36]. Both the resulting novel gene models and the annotated reference genes were included in expression quantification.

Gene-level raw counts were generated using featureCounts v1.5.0-p3 [37] in paired-end mode. Only properly paired, uniquely mapped fragments with a mapping quality of MAPQ ≥ 10 were counted; unpaired alignments, multimapping reads, and alignments with MAPQ < 10 were excluded. Fragments per kilobase of transcript per million mapped read (FPKM) values were calculated from gene-level raw counts, gene length, and the number of valid mapped fragments in each sample.

### 4.4. Expression Matrix Construction and Global Sample Analysis

Gene-level quantification results from all 42 samples were compiled into a raw-count matrix and an FPKM expression matrix, with genes as rows and samples as columns. FPKM values were transformed as log_2_ (FPKM + 1), and box plots were used to compare expression distributions among samples. Pairwise Spearman correlation coefficients were calculated using the cor function in R v3.5.0 (https://www.r-project.org/, accessed on 6 September 2026) and displayed as a heatmap. PCA was performed on the transformed expression matrix with samples as observations and genes as variables, and the first two principal components were visualized.

All 42 samples were included in expression matrix construction and global sample analyses. Because sampling was denser at the hpf time points and the principal analyses focused on development from 1 to 10 dpf, adjacent-stage differential expression analysis used the 27 samples from 1, 1.5, 2, 2.5, 3, 4, 5, 7, and 10 dpf. For WGCNA, the three biological replicates at each of these nine stages were averaged to generate nine stage-mean expression profiles.

### 4.5. Differential Expression Analysis Between Adjacent Developmental Stages

The 27 samples were compared between successive developmental stages. For each adjacent-stage comparison, genes with a summed FPKM greater than 1 across the six samples included in the comparison were retained, and the corresponding raw-count matrix was extracted. DESeq2 v1.20.0 [38] was used for normalization, dispersion estimation, and Wald testing, and the log_2_ fold change was calculated for the later stage relative to the preceding stage. Multiple testing was corrected using the Benjamini–Hochberg procedure [39]. Genes with |log_2_FC| ≥ 1 and a Benjamini–Hochberg-adjusted *p* value (*p*_adj_) ≤ 0.05 were considered significantly differentially expressed.

### 4.6. GO Enrichment and Functional Classification

GO [40] enrichment analysis was performed using the GO Enrichment function in TBtools-II v2.086 [41] for significantly upregulated genes from each adjacent-stage comparison and separately for genes in each WGCNA module. For adjacent-stage comparisons, the input set comprised significantly upregulated genes, and the background set comprised all GO-annotated genes that passed low-expression filtering and entered the corresponding DESeq2 test. For module-wise analysis, genes in each module constituted the input set, and the background comprised GO-annotated genes among the 12,185 genes included in WGCNA. Enrichment significance was evaluated using a hypergeometric test followed by Benjamini–Hochberg correction, with FDR ≤ 0.05 considered significant. The red module was selected for subsequent analysis after this uniform module-wise comparison because it contained the largest number of development-related GO terms. For Figure 3C, the same GO enrichment and redundancy-reduction procedures were applied to all adjacent-stage comparisons. Significant GO Biological Process terms were organized into the same broad functional categories using their GO names and definitions, and representative categories were displayed. Among enriched GO terms with an ancestor–descendant relationship, the corresponding sets of significantly upregulated genes were compared. When the ancestor term contained the same gene set as the descendant term or only one additional gene, the ancestor term was considered redundant and removed, whereas the more specific descendant term was retained. The reported term counts refer to the GO terms remaining after this procedure and were used to summarize functional composition rather than as independent events or a formal measure of change between intervals.

### 4.7. KEGG Pathway Mapping of Genes Upregulated at 3 dpf Relative to 2.5 dpf

For genes significantly upregulated in the 3 dpf vs. 2.5 dpf comparison, the NCBI Gene IDs corresponding to their LOC identifiers were submitted to KEGG Mapper–Color (https://www.kegg.jp/kegg/mapper/color.html, accessed on 6 September 2026). In the other-organisms mode, *L. reissneri* was specified using the organism code lrj. Matching genes were mapped onto the species-specific MAPK (lrj04010), Notch (lrj04330), TGF-β (lrj04350), and Wnt (lrj04310) pathway maps. Annotated *L. reissneri* gene nodes were shown in pale green, whereas successfully mapped upregulated genes were highlighted in magenta text.

### 4.8. Weighted Gene Co-Expression Network Analysis

Nine developmental stages (1, 1.5, 2, 2.5, 3, 4, 5, 7, and 10 dpf) were included. FPKM values were averaged across the three biological replicates at each stage, yielding nine stage-mean expression profiles. Genes with a stage-mean FPKM greater than 1 in at least one stage were retained, yielding 12,185 genes. A stage-level co-expression network was constructed using the WGCNA package in R [17]. Pairwise Pearson correlations were used to generate an unsigned weighted adjacency matrix. The pickSoftThreshold function was used to evaluate scale-free topology fit and mean connectivity for β values from 1 to 20, and β = 12 was selected for network construction. The resulting network was used to characterize broad temporal co-expression modules and prioritize candidate genes. 

The adjacency matrix was converted to an unsigned topological overlap matrix (TOM), and average-linkage hierarchical clustering was performed using 1 − TOM as the intergene distance. Initial modules were identified using Dynamic Tree Cut [18] with a minimum module size of 30 genes. Module eigengenes (MEs) were calculated, and modules with ME correlations greater than 0.75 were merged, corresponding to mergeCutHeight = 0.25. The gene dendrogram was plotted together with the initial Dynamic Tree Cut assignments and the merged module assignments.

A random subset of 1000 genes from the network was used to generate a TOM heatmap. Module clustering and a heatmap of module similarity were generated from Spearman correlations among MEs. Developmental stages were treated as categorical traits and represented by a stage-indicator matrix. Spearman correlation coefficients and associated *p* values were calculated for the relationships between each ME and each developmental stage and displayed in a module–stage relationship heatmap. These correlations were interpreted descriptively at the stage-mean level.

### 4.9. GO-Core Gene Screening and Intramodular Connectivity Analysis

Sixteen GO Biological Process terms directly related to embryonic development, germ-layer formation, and cell fate determination were selected from the terms significantly enriched in the red module. For each red-module gene, the number of selected terms to which it was annotated was calculated as a measure of recurrent functional annotation. Threshold sensitivity was evaluated at term-count thresholds of ≥2, ≥3, ≥4, and ≥5 by comparing the number of retained genes and their overlap with the top-100 high-connectivity gene set in the red module. Using all 597 red-module genes as the background, one-sided hypergeometric tests were applied to assess enrichment of the retained gene sets within the top-100 high-connectivity gene set. Considering both set size and overlap with highly connected genes, a term-count threshold of ≥4 was selected as the operational criterion for defining GO-core genes. Because this threshold was selected after comparison of the candidate thresholds, the resulting hypergeometric *p* value was treated as exploratory.

Intramodular connectivity (kWithin) was calculated from the WGCNA weighted network as the sum of connection weights between a gene and all other genes in the red module. The 597 red-module genes were ranked in descending order by kWithin, and the first 100 were designated the top-100 high-connectivity gene set. The overlap between GO-core genes and this set was quantified, and enrichment was evaluated by a one-sided hypergeometric test using all 597 red-module genes as the background.

Five development-related GO Biological Process terms and their associated genes were used to construct a GO term–gene relationship matrix, which was displayed as a chord diagram using the circlize package in R [42]. These five terms involved 44 unique genes. Using these genes as nodes, we extracted from the red-module edge list all edges for which both genes belonged to this set and the WGCNA connection weight was at least 0.02. The edges were imported into Cytoscape v3.10.3 [43] to construct a weighted subnetwork. The resulting network contained 44 nodes and 805 edges. Nodes represented genes, and edges represented co-expression connections. Node degree was calculated using Cytoscape NetworkAnalyzer and used to guide the network layout, with higher-degree nodes positioned toward the center and lower-degree nodes toward the periphery. Edge color intensity and width represented connection weight.

### 4.10. Identification and Temporal Profiling of Candidate Developmental Genes

Candidate genes were screened hierarchically using differential expression, co-expression network membership, functional annotation, and temporal expression profiles. First, GO-core candidate genes from the red module were combined with genes significantly upregulated in the 3 dpf vs. 2.5 dpf comparison (log_2_FC ≥ 1 and *p*_adj_ ≤ 0.05), and duplicate entries were removed to generate an initial candidate set. Genes were then retained if their annotations were related to germ-layer formation, axial patterning, cell fate determination, neural development, organogenesis, cell migration, or morphogenesis, or if they belonged to developmental regulatory families including Wnt, TGF-β/BMP, Notch, FGF, and Hox. Canonical regulatory genes not represented in these sets but with established conserved functions in previous studies of vertebrate embryogenesis were also included as candidates. This procedure compiled a candidate list based on existing functional annotations for subsequent studies; the functions of individual genes remain to be tested experimentally.

Standardized temporal expression profiles were generated from the mean expression of the three biological replicates at each stage from 1 to 10 dpf. Candidate genes were divided into three clusters according to the timing of their main expression increase and their subsequent expression trajectory: an increase around 2.5–3 dpf followed by a decline, an increase beginning in this interval followed by sustained expression, and a progressive increase at later stages. Within-gene Z-scores were used only to compare the relative expression of each gene across developmental stages.

### 4.11. RT-qPCR Validation

Eight representative genes were selected for RT-qPCR based on differential expression, functional annotation, red-module membership, and temporal expression pattern, covering genes that increased around 2.5–3 dpf, remained highly expressed thereafter, or increased during later development.

Samples for RT-qPCR were collected at 1, 2, 2.5, 3, 4, 5, 7, and 10 dpf using the embryo culture, developmental staging, and sampling criteria described above. For each biological replicate, 50 embryos at comparable developmental stages were pooled, immersed in 1 mL RNAiso Plus (TaKaRa Bio, Kusatsu, Japan), snap-frozen in liquid nitrogen, and stored at −80 °C.

Total RNA was extracted using RNAiso Plus in combination with RNA Extraction Assisted Reagent (REX; Accurate Biology, Changsha, China) and high-salt isopropanol precipitation. cDNA was synthesized from 2 μg total RNA in a 20-μL reaction using the Evo M-MLV RT Premix for qPCR Ver.2 (Accurate Biology, Changsha, China). Reverse transcription was performed at 37 °C for 15 min, followed by 85 °C for 5 s.

RT-qPCR was performed on an ABI 7500 Real-Time PCR System (Applied Biosystems, Foster City, CA, USA) using 2× SYBR Green Pro Taq HS Premix (ROX plus; Accurate Biology, Changsha, China). Each 20-μL reaction contained 10 μL of 2× premix, 0.4 μL each of the forward and reverse primers (10 μM), and 9.2 μL of diluted cDNA (adjusted to 10 ng RNA-equivalent/μL with nuclease-free water). The amplification program consisted of 95 °C for 30 s, followed by 40 cycles of 95 °C for 5 s and 60 °C for 30 s, with melting-curve analysis performed after amplification. Amplification efficiencies were evaluated using standard curves generated from fivefold serial dilutions of cDNA at template concentrations of 10, 2, 0.4, 0.08, and 0.016 ng RNA-equivalent/μL. All target and reference primer pairs exhibited amplification efficiencies of 92–105%, with coefficients of determination (R^2^) greater than 0.99. Three biological replicates were analyzed at each developmental stage, with three technical replicate wells for each biological replicate. The mean Ct value of the three technical replicates was used for subsequent analysis.

LOC133343421 (*ATP5MK*) was selected as the reference gene based on its mean expression, temporal stability in the RNA-seq dataset, and primer specificity. Its stability in the RT-qPCR samples was further assessed from the mean, range, and coefficient of variation in the Ct values across the 24 biological samples. Relative expression was calculated using the 2^−ΔΔCt^ method [44], with the 1 dpf samples used as the calibrator. Primers were designed using NCBI Primer-BLAST web tool (https://www.ncbi.nlm.nih.gov/tools/primer-blast/; accessed on 2 October 2026) [45] and synthesized by GenScript Biotech Corporation (Nanjing, China); their sequences are provided in Supplementary Table S4.

## 5. Conclusions

We generated a continuous RNA-seq resource comprising 14 developmental stages from 2 hpf to 10 dpf in *L. reissneri*. The time series describes progressive changes in gene expression and functional composition across early development. Among these stage-associated patterns, the 2.5–3 dpf interval showed broad enrichment of genes related to embryonic patterning and morphogenesis. Stage-level co-expression analysis and functional annotation organized these expression patterns into module and candidate-gene contexts, and RT-qPCR reproduced the main temporal expression trends of eight representative genes. The dataset provides a temporal framework for subsequent morphological, spatial-expression, and functional studies of early lamprey development.

## Figures and Tables

**Table 1:** Sampling times, corresponding developmental stages, and degree-day values for *L. reissneri* embryos at the nominal incubation temperature of 18 °C. The degree-day values were calculated as 18 °C multiplied by developmental time and are provided as an aid for comparison.

Sampling Time	Degree-Days at 18 °C (°C·d)	Corresponding Developmental Stage
2 hpf	1.5	Fertilization
8 hpf	6	Cleavage (2-cell)
12 hpf	9	Cleavage (8-cell)
18 hpf	13.5	Cleavage (64-cell)
24 hpf (1 dpf)	18	Early blastula
30 hpf (1.25 dpf)	22.5	Blastula
1.5 dpf	27	Blastula
2 dpf	36	Blastula
2.5 dpf	45	Late blastula
3 dpf	54	Early gastrula
4 dpf	72	Gastrula
5 dpf	90	Neurula
7 dpf	126	Head protrusion
10 dpf	180	Hatching

## Data Availability

The raw RNA-seq data have been deposited in the NCBI Sequence Read Archive under BioProject accession number PRJNA1503392. Supplementary Tables S1–S4 provide sequencing quality-control and read-alignment statistics, GO-core genes and functional annotations for the red module, candidate developmental genes associated with pronounced transcriptional changes from 2.5 to 3 dpf, and RT-qPCR primer sequences with corresponding gene names.

## Supplementary Materials

The following supporting information can be downloaded at https://www.mdpi.com/article/10.3390/ijms27209001/s1.
