# Supplementary material for: A Time-Course Transcriptomic Resource for Early Embryogenesis in Lethenteron reissneri
Source: Int J Mol Sci. 2026 Oct 11;27(20):9001. doi: 10.3390/ijms27209001 (PMC13705178; doi:10.3390/ijms27209001)
Supplement: Supplementary file 1 [file ijms-27-09001-s001.zip › Supplementary Figure S3.pdf]

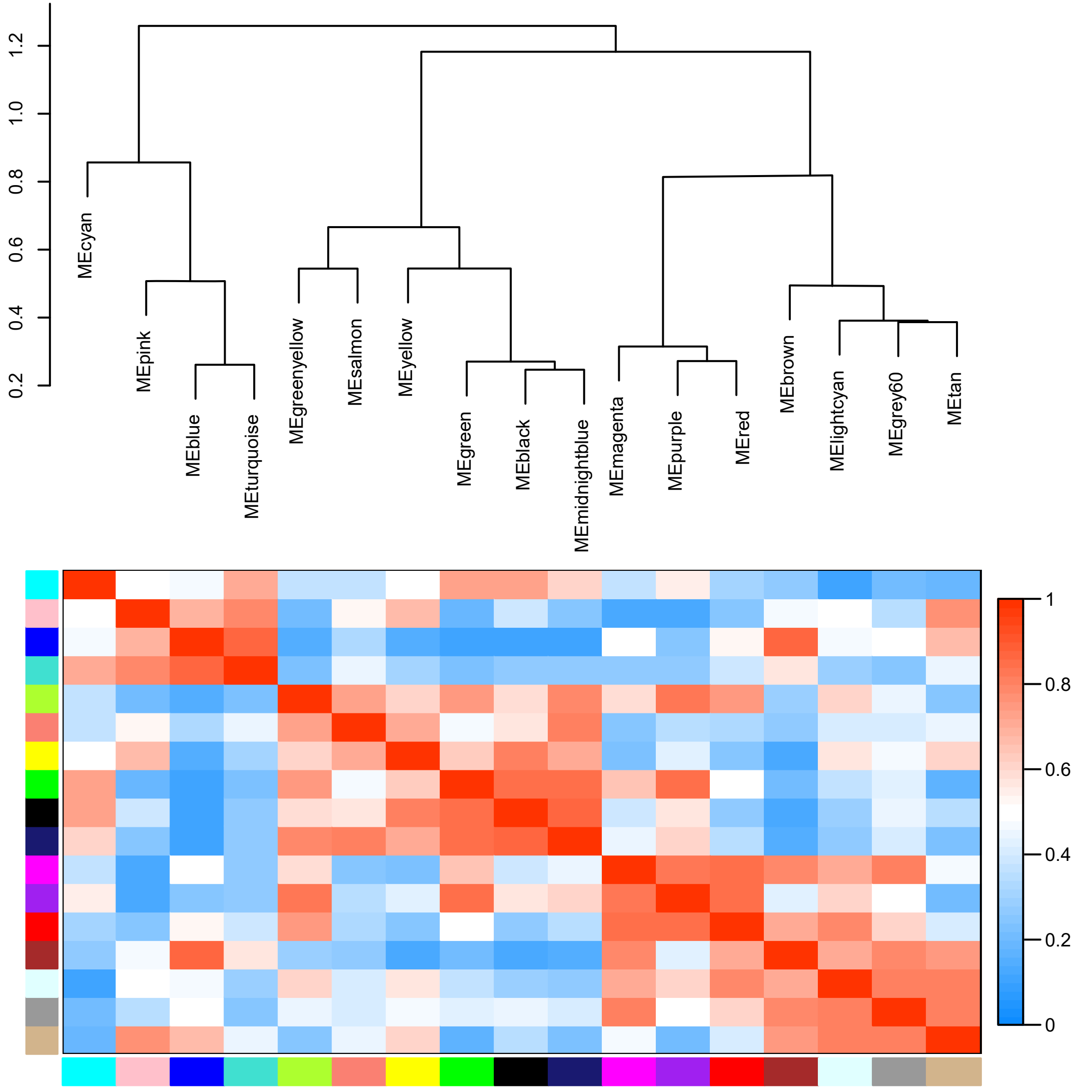

1.2
1.0
0.8
0.6
0.4
0.2
MEcyan
MEpink
MEblue
MEturquoise
MEgreenyellow
MEsalmon
MEyellow
MEgreen
MEblack
MEmidnightblue
MEmagenta
MEpurple
MEred
MEbrown
MElightcyan
MEgrey60
MEtan
1
0.8
0.6
0.4
0.2
0
